# Accessory Extraocular Muscle: A Rare Cause of Strabismus

**DOI:** 10.5334/jbsr.3807

**Published:** 2024-11-30

**Authors:** Ana Carolina Chaves, Catarina Paiva, Sílvia Carvalho

**Affiliations:** 1 Neuroradiology Unit, Medical Imaging Department, Coimbra Local Health Unit, Coimbra, Portugal; 2 Pediatric Ophthalmoloy, Department of Ophthalmology, Coimbra Local Health Unit, Coimbra, Portugal; 3 Faculty of Medicine of the University of Coimbra, Coimbra, Portugal

**Keywords:** Accessory extraocular muscles, strabismus, pediatric ophthalmology, orbit, CT, MRI

## Abstract

*Teaching point:* Accessory extraocular muscles are rare intraorbital congenital structures that can cause diplopia and restrictive strabismus.

## Case Presentation

**Figure 1:**
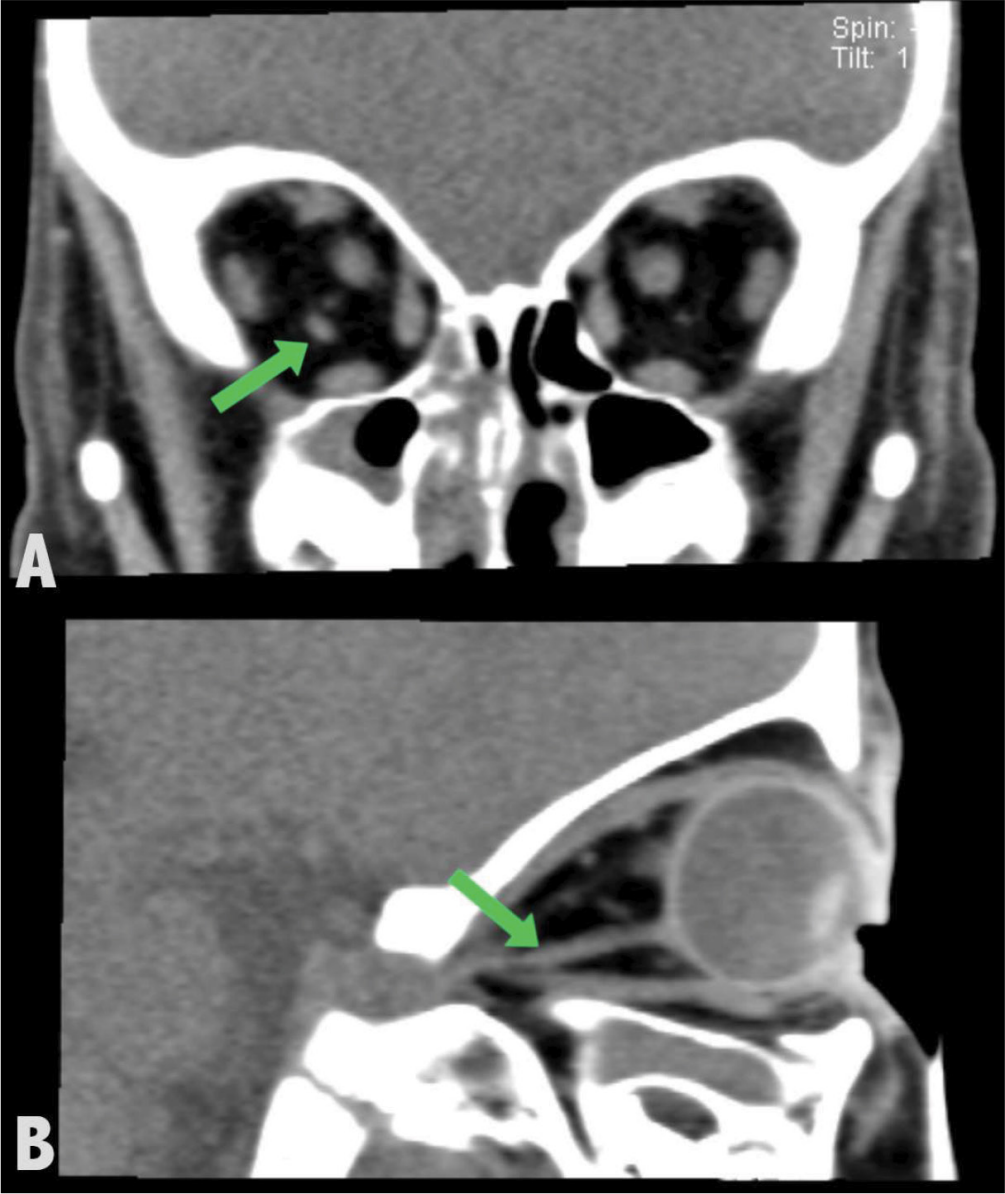
Unenhanced coronal (**a**) and sagittal (**b**) CT images of the orbits reveal a right accessory extraocular muscle (green arrows) located inferolateral to the optic nerve, medial to the lateral rectus muscle, and superior to the inferior rectus muscle. The muscle exhibits a density similar to that of the other extraocular muscles.

**Figure 2:**
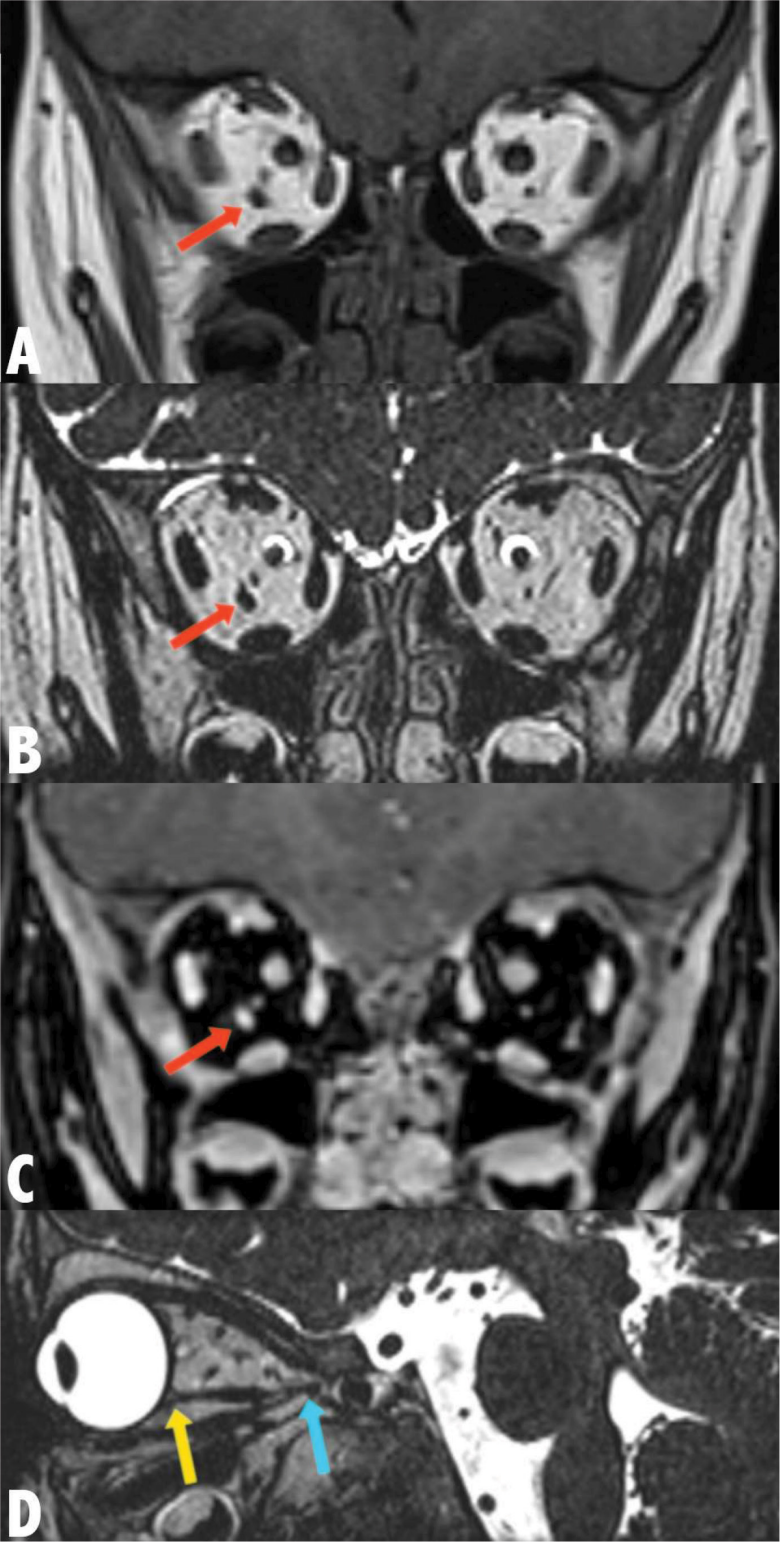
MRI Coronal T1‑WI (**a**) and coronal T2‑WI (**b**) of the orbits demonstrate an accessory extraocular muscle in the right orbit (red arrow), appearing isointense to other extraocular muscles. Coronal post‑gadolinium T1‑WI with fat saturation (**c**) reveals the accessory extraocular muscle (red arrow) with increased signal intensity, consistent with other extraocular muscles. Sagittal T2‑WI (**d**) of the right orbit shows the accessory extraocular muscle attaching anteriorly to the posterolateral aspect of the globe (yellow arrow) and posteriorly the annulus of Zinn (blue arrow).

A 3‑year‑old boy presented to the emergency department with a one‑month history of difficulty elevating his right eye. His visual acuity was 20/20 in each eye, and the red reflex test was normal. However, upon up‑gaze, he demonstrated an absence of elevation in his right eye along with enophthalmos. A computed tomography (CT) scan revealed an anomalous longitudinal structure measuring 2.3 mm in diameter located in the right intraconal space, with a density equal to that of the extraocular muscles (Figure 1). Magnetic resonance imaging (MRI) confirmed a well‑defined, linear solid structure in the right intraconal space, originating from the annulus of *Zinn*, coursing inferolaterally to the optic nerve, and inserting on the posterior surface of the globe. This structure appeared isointense to the extraocular muscles on T1‑ and T2‑weighted images (WI), with homogeneous enhancement resembling that of the extraocular muscles (Figure 2), thus leading to a diagnosis of an accessory extraocular muscle. Several follow‑up appointments with a pediatric ophthalmologist were scheduled to monitor the patient's symptoms. As the child grew taller, the limitation of elevating his right eye became significantly less disruptive to his daily activities, and therefore, a conservative approach was considered the best option for this case, given the potential complications associated with surgery.

## Discussion

**Figure 3:**
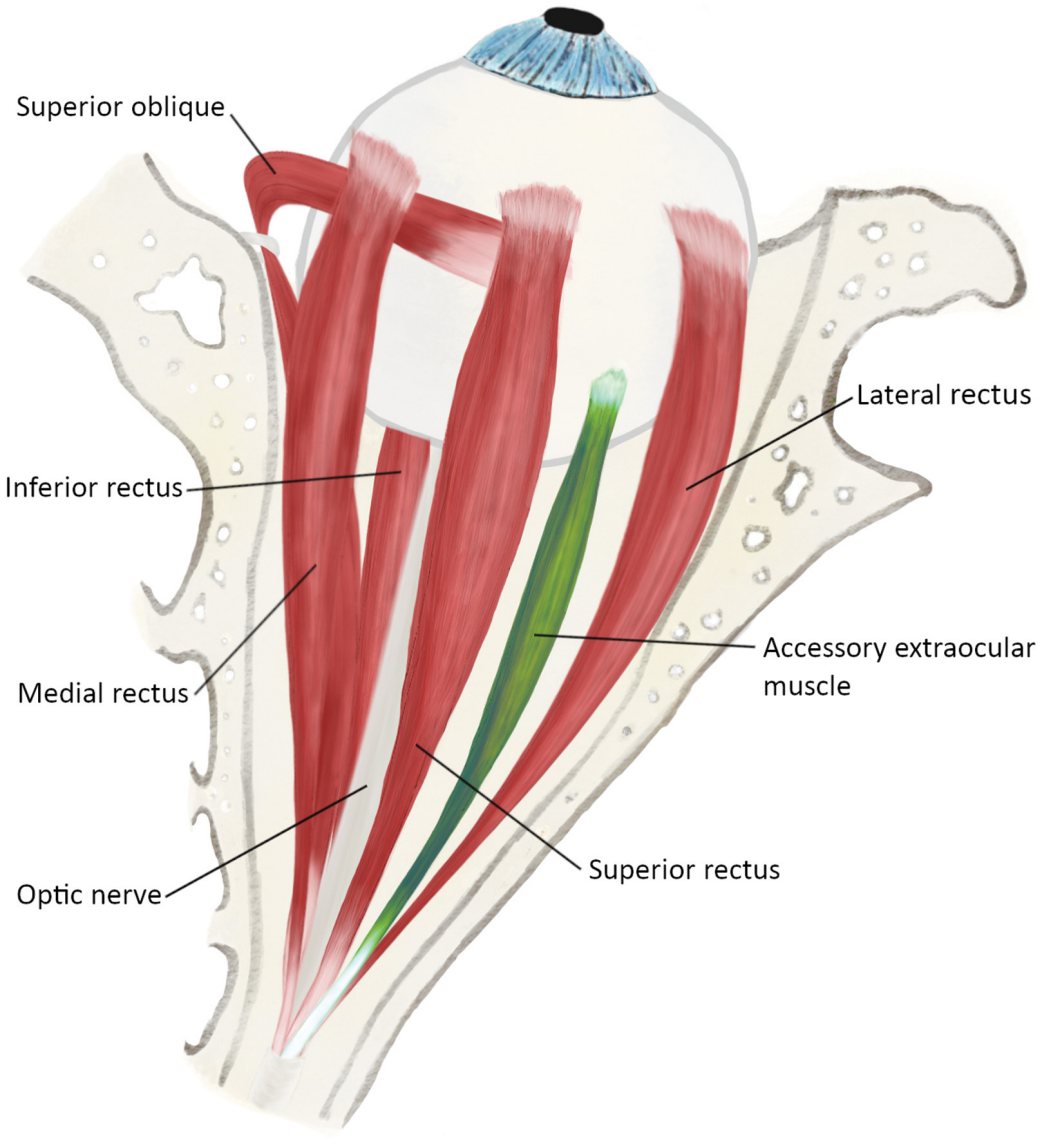
A superior anatomical view of the extraocular muscles is shown (excluding the inferior oblique muscle), with the anomalous accessory extraocular muscle highlighted in green. This illustration was created by Ana Carolina Chaves, the first author, in 2023.

Humans have six extraocular muscles that control both voluntary and involuntary eye movements. These muscles include the superior rectus, superior oblique, inferior rectus, inferior oblique, medial rectus, and lateral rectus (Figure 3). Accessory extraocular muscles are rare anomalous orbital structures that can interfere with eye movement and lead to motility disorders (Figure 3), resulting in symptoms such as diplopia and strabismus [1]. Their exact prevalence is unknown, as only a few cases have been reported in the literature [1]. Three types of accessory extraocular tissues have been described: first, muscular fibers arising from the original extraocular muscle and attaching to abnormal locations; second, a fibrous band located beneath the extraocular muscle and following its trajectory; and third, an isolated extraocular muscle originating from the annulus of *Zinn* and inserting on the posterior globe [1]. This case represents the third type. One hypothesis is that this isolated accessory extraocular muscle corresponds to an ancestral retractor bulbi muscle [1]. While this muscle is absent in *Homo sapiens*, it is commonly found in reptiles, amphibians, and ruminants, originating at the orbital apex and inserting on the posterior surface of the ocular globe. Its function is to serve as a protective mechanism, retracting the ocular globe in response to pain or threat, and, in amphibians, it plays a role in swallowing. This case exemplifies a rare anomalous intraorbital congenital structure that presents with diplopia and restrictive strabismus. Despite their rarity, it is important to consider the possibility of these developmental anomalies. This awareness is crucial for accurate identification, otherwise, these anomalies may go unnoticed.
